# Development of Flat Silicon-Based Mesh Lens Arrays for Millimeter and Sub-millimeter Wave Astronomy

**DOI:** 10.1007/s10909-019-02327-y

**Published:** 2020-01-24

**Authors:** Giampaolo Pisano, Jason Austermann, James Beall, Nils Halverson, Johannes Hubmayr, Gregory Jaehnig, Christopher M. McKenney, Benjamin Raymond, Aritoki Suzuki

**Affiliations:** 1 School of Physics and Astronomy, Cardiff University, Cardiff, UK; 2 National Institute of Standards and Technology, Boulder, CO USA; 3 University of Colorado, Boulder, CO USA; 4 Lawrence Berkeley National Laboratory, Berkeley, CA USA

**Keywords:** Quasioptical systems, Millimeter waves, Optical coupling, Focal plane arrays, Cosmic microwave background

## Abstract

The high sensitivity requirements set by future cosmic microwave background instruments are pushing the current technologies to produce highly performant focal plane arrays with thousands of detectors. The coupling of the detectors to the telescope optics is a challenging task. Current implemented solutions include phased-array antenna-coupled detectors, platelet horn arrays, and lenslet-coupled planar antennas. There are also recent developments of flat graded-index lenses based on etched silicon. However, there are strong requirements in terms of electromagnetic performance, such as coupling efficiency and bandwidth, as well as requirements in terms of easy manufacturing and scalability, and it is very challenging to meet all these requirements with one of the above solutions. Here, we present a novel approach for producing flat metal-mesh lenslet arrays based on devices previously realized using the mesh-filter technology. We have now adapted the polypropylene-based mesh lens design to silicon substrates, thus providing a good mechanical match to the silicon-based detector arrays. The measured performance of prototype pixels operating at millimeter wavelengths is presented.

## Introduction

It is now clear that future cosmic microwave background (CMB) experiments, both ground and space based, will need broad spectral coverage to reach an all new level of sensitivity and systematics control in order to detect any potential inflationary signal and to enter a precision measurement era for B-mode signals.

Space-based (sub-)millimeter and far-IR experiments have provided paradigm shifting results in astrophysics. For example, in the study of star formation, herschel showed that protostellar cores form within molecular filaments [1] and planck is giving us the first real look at the scale and scope of dust polarization in our galaxy [2]. These observatories, together with sub-orbital and ground-based experiments, have also contributed to a wide range of astrophysical fields of study, including star-formation history and high-redshift galaxies (e.g., [3]), the role of magnetic field in star formation (e.g., [4]), galaxy cluster dynamics and cataloguing via the thermal Sunyaev–Zel’dovich (SZ) effect (e.g., [5]), bulk flow and cluster dynamics via the kinetic SZ effect [6–8], tests of patchy reionization through the kinetic SZ effect [9], and even the relatively unexplored discovery space for transient millimeter and far-IR sources (e.g., [10–12]). Space-borne experiments have traditionally produced some of the richest and most unique data sets in these research areas due to their access to wavelengths that are inaccessible from the ground and their ability to produce unbiased all-sky maps at the largest angular scales. Future discoveries in the far-IR and millimeter will undoubtedly leverage future space missions and the new technologies that enable them.

The planar, metamaterial optical coupling technologies being developed and reported here may directly enable the large, compact, and robust detector arrays required for future astrophysical experiments. This includes potential applicability to many far-IR projects currently in development or consideration, such as the long-wavelength components of the Origins Space Telescope (OST; formerly Far-IR Surveyor) [13], the Galactic Evolution Probe (GEP) [14], and the potential sub-orbital experiment BFORE [15].

## Lenslet Array Current Technologies

**Fig. 1:**
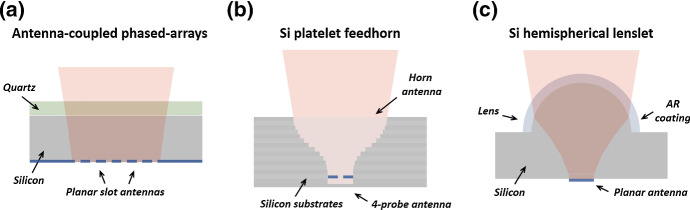
Schematic of several existing technologies for focusing radiation on to a detector. **a** Antenna-coupled phased arrays, **b** Si platelet feedhorns, **c** Si hemispherical lenslets (color figure online)

To meet the challenge of efficiently coupling telescope optics to CMB detectors, a variety of technologies have been developed. These have been driven by requirements for beams that are Gaussian with low side lobes, low cross-polarization, and low cross-talk. Additional requirements include sufficient bandwidths, scalability, and easy manufacturing. Three currently competitive technologies are phased-array antenna-coupled detectors, platelet feedhorn arrays, and lens-coupled antennas (Fig. 1).

Planar phased arrays offer an elegant solution where the beams are synthesized by coherently emitting slot antennas [16, 17]. The antennas, the filters, and the detectors are all fabricated with the same photolithographic processes. Ratio of main lobe to side lobe amplitude can be adjusted by designing how sub-arrays are illuminated. Both uniform illumination that results in high side lobe and Gaussian illumination for Gaussian beam have been demonstrated. So far, a single frequency version of the technology has deployed successfully in multiple CMB experiments. A multichroic version of the technology is in development.

Feedhorn technology has been used by many CMB experiments over decades. Silicon platelet feedhorns have been developed using photolithography. Circular apertures are etched in silicon wafers which are then stacked together and eventually copper- and gold-plated. Corrugated feedhorns have achieved bandwidths of 2.3:1 [18, 19]. Spline-profiled smooth-wall feedhorns, used in several current and upcoming ground-based experiments [20, 21], are easier to fabricate and offer increased filling factor of the focal plane.

Lens-coupled antenna architecture has been used in millimeter and sub-millimeter applications such as POLARBEAR, SPT-3G, and DESHIMA [22]. Hyper-hemispherical, elliptical, or synthesized elliptical lenses are placed on the top of planar antennas to increase their forward gain and their optical coupling efficiency to the telescope. For millimeter and sub-millimeter wave applications, high dielectric constant and low loss materials, such as silicon and alumina, have been used as lens materials. Matching layers (also known as anti-reflection coatings) are often applied to minimize reflection at vacuum–dielectric interface. Planar antennas, such as double slot dipole, sinuous, and leaky antenna have been used.

The lens-coupled antenna architecture has several desirable properties. The observation frequency bandwidth of a lens-coupled detector can be designed to be wider than other optical coupling technologies, and it is limited by either the antenna’s or the anti-reflection coating’s bandwidth. Recent CMB experiments have implemented broadband lens-coupled antenna detectors by developing broadband planar antenna and broadband anti-reflection coating techniques. The ability to couple to a planar antenna allows microfabrication techniques to produce antenna and sensors on a silicon wafer. A lens array can be fabricated separately from the wafer holding the sensors. The two can be aligned and mated together to form an optically active detector.

Multiple methods have been used to fabricate lens arrays with matching layers. Deployed CMB experiments, such as POLARBEAR and SPT-3G, used dielectric hemispheres placed in pockets that are micromachined into silicon wafer with DRIE techniques.

Both millimeter and sub-millimeter experiments explored machining the anti-reflection coated lens array from an ingot of silicon. For sub-millimeter experiments, gray-scale lithography has also been used to make lens arrays on silicon wafers.

## Metamaterial Lenslet Array Developments

**Fig. 2:**
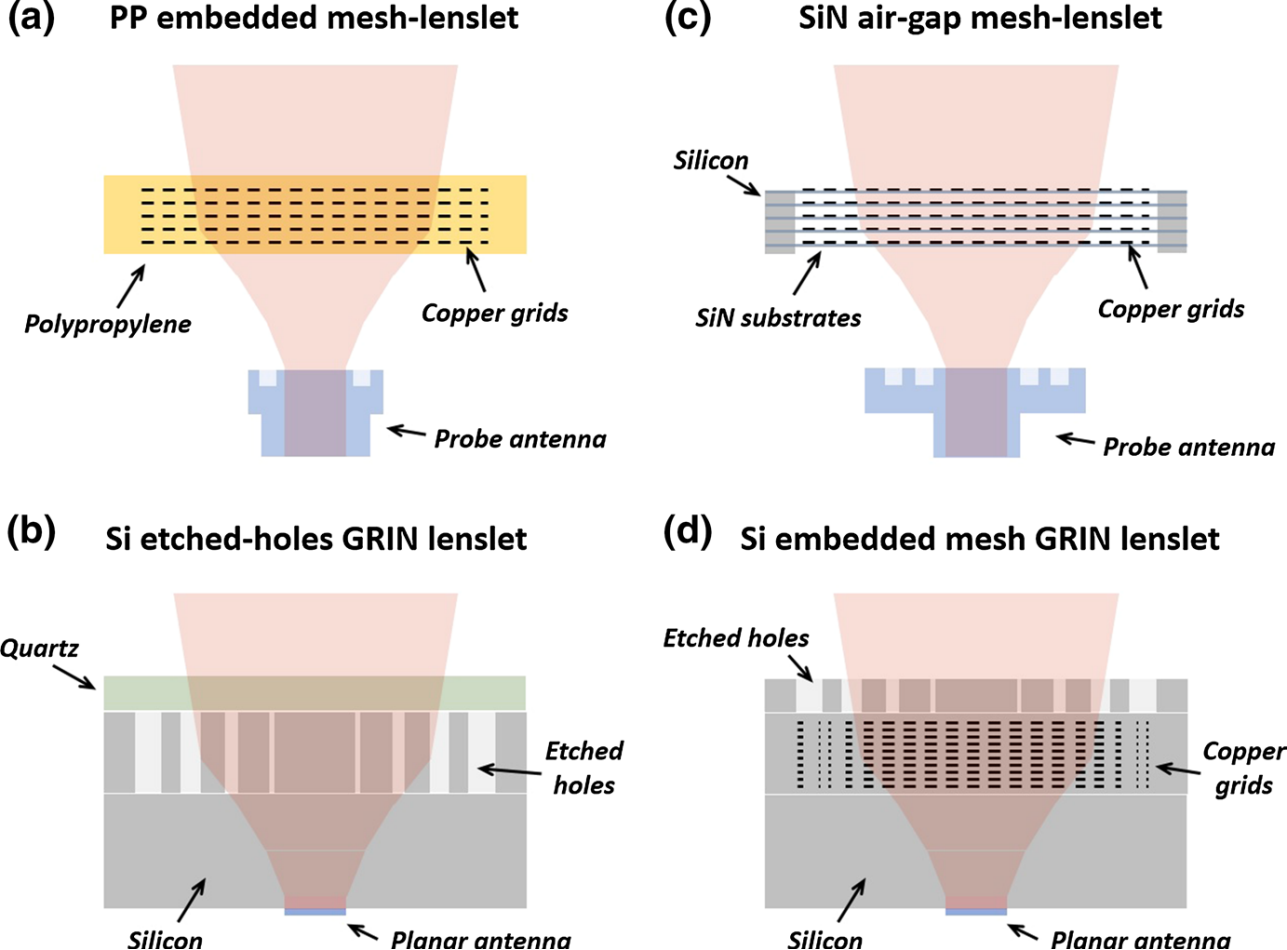
Four types of lenslet arrays based on metamaterials. **a** In the polypropylene-embedded mesh lenslet approach, metal squares are deposited directly on to polypropylene sheets. These grids act as transmission line elements and shape the beam exiting the waveguide. **b** The Si-etched-holes GRIN lenslet is based on a gradually decreasing refractive index which refocuses the beam. **c** The SiN air-gap mesh lenslet uses free floating metal-mesh elements fabricated on an SiN membrane to generate a lens. **d** The Si-embedded mesh GRIN lenslet is based on a gradually increasing index obtained embedding metal squares into the silicon (color figure online)

In this section, we will describe our development of lenslet arrays based on metamaterials. Two completely different approaches, based on different technologies, were independently developed: the polypropylene-embedded mesh lenslets (Fig. 2a) and the silicon-etched-holes gradient refractive index (GRIN) lenslets (Fig. 2b). The working principle of the first was then implemented using the technology of the second, and SiN air-gap mesh lenslets were successfully demonstrated (Fig. 2c). More recently, a Si-embedded mesh GRIN lenslet directly coupled to an antenna has been developed (Fig. 2d).

### Polypropylene-Embedded Metal-Mesh Phase-Delay Lenslets

The mesh technology has been employed for decades to fabricate filters at millimeter and sub-millimeter wavelengths [23]. Recent developments include mesh half-wave plates, flat mesh lenses, and mesh lenslet arrays [24–27]. All these designs rely on copper grids embedded into polypropylene, manufactured using photolithographic techniques. The grids behave like transmission lines that, in the case of the lenses, can be locally designed to provide the transmissions and the phases required to mimic the behavior of a classical lens. The design is performed using specifically written codes which are then verified using finite-element analysis (HFSS). Flat mesh lenslet arrays operating within the whole W-band (75–110 GHz) have been demonstrated and reported elsewhere [27]. They are designed to work in free space: The radiation from the lenses is coupled to the detectors via waveguide probe antennas (see Fig. 2a). The coupling of this type of arrays to Si-based detectors operating at cryogenic temperatures is not straightforward due to the differential thermal contractions that have to be taken into account and compensated.

### Silicon-Etched GRIN Lenslets

An effective index can be achieved in by etching holes on a sub-wavelength pitch in a dielectric material such as Si. By radially varying the diameter of the holes, an effective gradient radial index is achieved and a gradient refractive index (GRIN) lens can be formed (Fig. 2b). In our approach, the lens is fabricated from Si wafers and directly coupled to the backside of an antenna-coupled detector, allowing more than 90% of the radiation to be transmitted from antenna to lenslet without the use of a backshort. Fabrication from Si and the use of planar anti-reflection materials eliminate alignment problems which might arise from dissimilar material coefficients of thermal expansion (CTE).

**Fig. 3:**
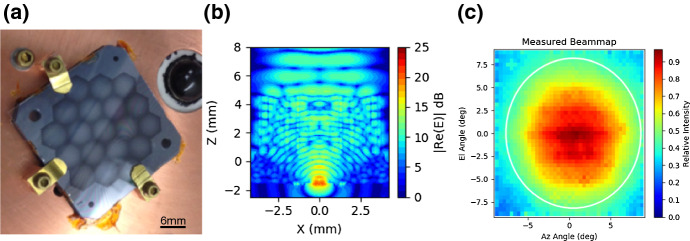
Silicon-etched GRIN lenslet. **a** Fabricated 19-element lenslet sub-array mounted next to an existing single-pixel hemispherical lens. The GRIN lenslet comprises a stack of 540-μm-thick Si wafers with etched holes generating sub-wavelength effective indices. **b** Cross section of the electric field simulated in HFSS. A sinuous antenna at the origin launches a Gaussian beam which is intercepted by the lens and collimated before exiting free space via a quartz anti-reflection layer. **c** Measured optical beam map of a silicon-etched GRIN lenslet using a sinuous antenna-coupled TES with band center ~ 150 GHz. Measured FWHM (white circle) is 16.3°, near the HFSS simulation value of 17.0° (color figure online)

We developed a prototype lenslet array for operation at the 90 and 150 GHz bands as shown in Fig. 3a. The lenslet was simulated with finite-element analysis software—HFSS (Fig. 3b). Preliminary beam maps were obtained as shown in Fig. 3c. Measurements and simulations were in good agreement with a full width at half maximum (FWHM) of 17.0°. Simulations of the lenslet show losses (~ 20%) into the Si substrate which are caused primarily by the low angle of total internal reflection. There are approaches which can mitigate this, but they are computationally intensive and we have not yet extensively pursued them.

This approach is promising for long-wavelength ($\lambda \gtrsim 2 \; {\text {mm}}$) applications. However, for shorter wavelengths where a smaller pitch is required to keep features in the sub-wavelength regime, fabrication constraints become problematic. Achieving the same index with a smaller pitch reduces the volume of Si remaining between two adjacent holes. We have successfully fabricated devices with 40-μm distance between the edges of nearest neighbor holes which is sufficient for operation to ~ 180 GHz, but using thinner Si between holes introduces challenges with both fabrication and mechanical robustness.

### Air-Gap Metal-Mesh Phase-Delay Lenslets

**Fig. 4:**
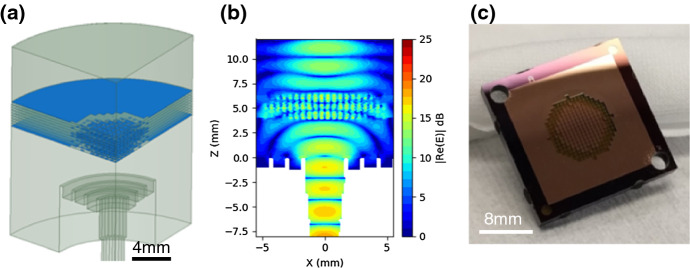
Initial air-gap metal-mesh phase-delay lenslet prototype design. **a** Finite-element model details showing the mesh lenslet (blue layers) coupled via free space to a circular horn antenna (gray at bottom). **b** Cross section of the E field from an HFSS simulation of the lens, showing the curved wavefront exiting the probe before being collimated by the lens. **c** Photograph of the fabricated prototype lenslet (color figure online)

**Fig. 5:**
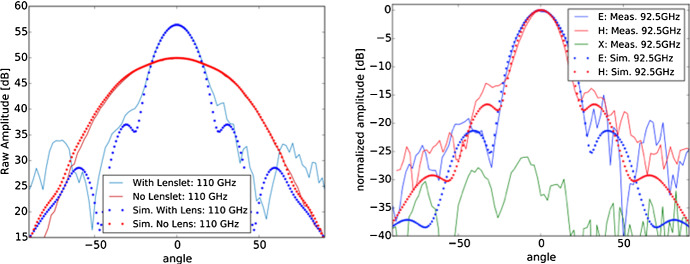
Measurements (solid lines) of the air-gap metal-mesh phase-delay lenslet prototype compared to simulation (data points). Left: Forward gain of device at 110 GHz as illustrated through the results of our VNA measurements (see text) with and without the lenslet. Simulations are peak normalized to the measurements. Right: E- and H-plane measurements of the lenslet at 92.5 GHz compared to simulation. Also shown are the cross-polarization (*X*) measurements, which are believed to be dominated by alignment-related systematic errors specific to the measurement, thus providing an approximate upper limit that is within desired tolerance (color figure online)

Our first attempt to transfer the polypropylene metal-mesh phase-delay technology into the silicon technology consisted of using 2-μm-thick SiN membranes supporting the metal grids (see Fig. 2c). The membranes were supported by silicon frames at their edge. In order to simplify the manufacturing processes, it was possible to design the mesh lens with equi-spaced membranes, at 250-μm distance from each other. The mesh lens was optimized to be coupled to a commercial waveguide probe antenna, which was then connected to a vector network analyzer for its tests. The SiN air-gap mesh lenslet was designed to operate in W-band. The finite-element model, an example of electromagnetic simulations, and a manufactured prototype are all shown in Fig. 4. The experimental characterization of the SiN mesh lens provided results very close to the expected performance. The narrowing of the beam can be seen by comparing the beam of the probe and that obtained adding the mesh lens in front of it (see Fig. 5a). Examples of modeled and measured co-polar and cross-polar beams are reported in Fig. 5b.

### Silicon-Embedded Metal-Mesh GRIN Lenslets

The silicon-embedded metal-mesh GRIN lenslet was developed to efficiently couple incident radiation from a broadband sinuous antenna while using a fabrication approach with pitch limited only by traditional photolithography techniques, enabling scaling to higher frequencies. In this approach, a GRIN lenslet is formed from metal squares patterned on stacked Si substrates to synthesize a GRIN lenslet with higher indices than the native Si. This higher index GRIN lenslet is coupled to the backside of a sinuous antenna on Si. Light is collimated within the lens and exits via a planar anti-reflection coating to air. A planar anti-reflection coating is made by etching holes in Si and is easily extensible to broad bands by changing the hole diameters.

**Fig. 6:**
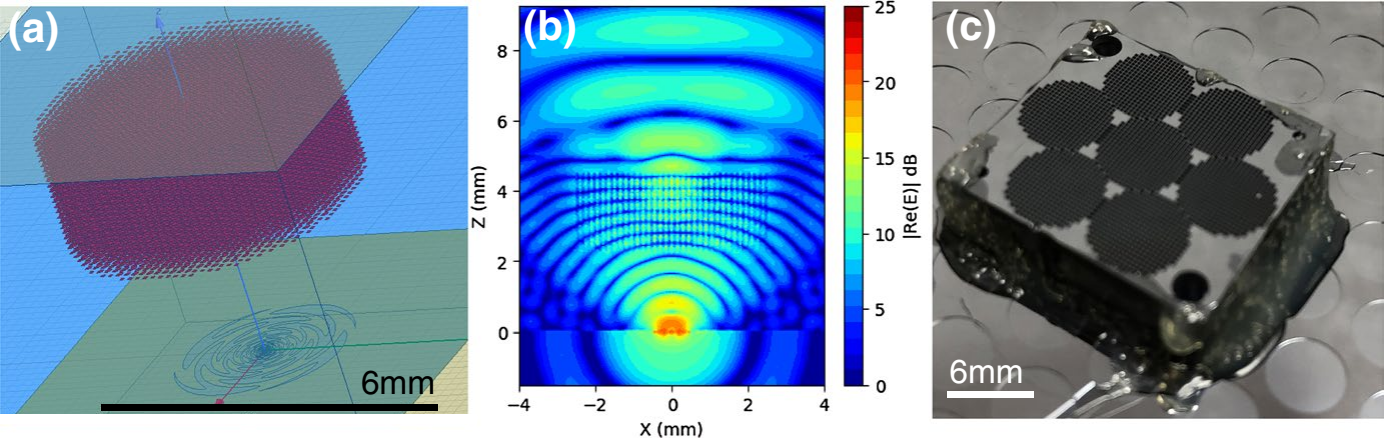
Silicon-embedded metal-mesh GRIN lenslet. **a** Ansys HFSS model. The sinuous antenna is visible on the bottom of the Si (blue) substrate. The silicon-embedded metal-mesh GRIN (metal-mesh squares shown in red) is separated from the antenna by 2 mm of silicon, including that of the detector wafer. **b** Electric field in cross section. A Gaussian beam is launched from the antenna at the origin and intercepted by the metal-mesh squares which collimate the beam. It exits the Si to free space via a planar anti-reflection layer. **c** Fabricated lenslet sub-array installed on a PB/SA 90/150 GHz focal plane. Visible on top are the anti-reflection layers made by etching holes in silicon wafers (color figure online)

A prototype lenslet array was designed by first using a semi-analytical equal-time approach and then simulating and optimizing the structure in Ansys HFSS. The structure consists of a mesh grid with a 125-μm pitch filling 5 mm of the available 6.8 mm pixel pitch, with 34 total mesh layers separated by 100 μm in the full lenslet stack. This is shown schematically in Fig. 6a with a cross section of E field from the HFSS simulation shown in Fig. 6b. The smaller mesh diameter was chosen to allow simulations to converge in reasonable (less than one week) time scales using available computational tools. Simulations of single-cell elements suggest that 100 MB order of memory is needed per metal-mesh element to sufficiently converge the electromagnetic parameters. We have recently gained access to a research cluster with 4 TB of memory and shown better convergence in models. However, we have not yet utilized this to fully optimize a model.

The first version of silicon-embedded metal-mesh GRIN lenslet test sub-arrays, shown in Fig. 6c, was fabricated from 150-mm-diameter silicon-on-insulator (SOI) wafers with a 100-μm device layer bonded to a 520-μm handle wafer. Each wafer yielded 30 separate 19 mm × 20 mm platelets containing 7 lenses in a hexagonal array. The lens platelets were fabricated by electron beam evaporating 400 nm of copper on a liftoff photoresist pattern, deep reactive ion etching the platelet outline pattern and then removing the handle wafer by deep reactive ion etching from the reverse side. For subsequent silicon-etched GRIN lenslet arrays, we have developed fabrication processes to directly handle 100-μm wafers.

**Fig. 7:**
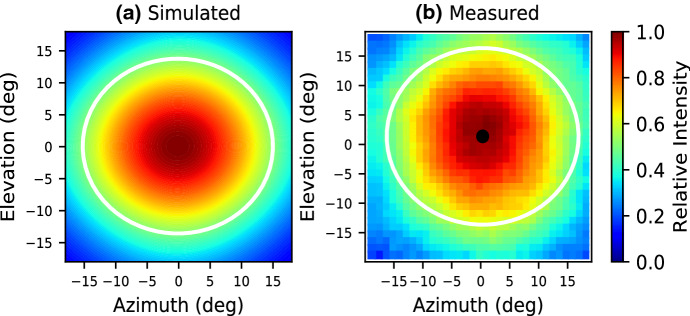
3D beam map of the silicon-embedded metal-mesh GRIN . **a** Simulated beam map of lenslet from simulations in HFSS at 90 GHz. The white ellipse is a fit to the FWHM. **b** Map made using our 6-degree-of-freedom (6 dof) beam mapper, which allows the source to point directly at the detector from arbitrary angles. The white ellipse is a fit to the measured FWMH (color figure online)

Measurements were carried out by mounting this sub-array directly to a Polarbear-2 focal plane and using a 6-degree-of-freedom beam mapper to characterize the optical response, allowing a fully three-dimensional beam map to be measured and compared to simulation (Fig. 7). The measured beam fits an ellipse with FWHM of 33.2° and 30.5° along the major and minor axes, respectively, whereas the simulated lenslet was narrower with FWHM of 30.8° and 28.0°, respectively. The wider beam may be due in part to the lower frequency of this particular plane with a frequency center of 85 GHz due to a fabrication error, whereas our simulations were carried out assuming a frequency of 90 GHz. Additionally, there has always been discrepancy between measured and simulated beams with hemispherical lenslets as well, and we are investigating sources of these discrepancies.

## Conclusions

The optical coupling is one of the technological challenges currently faced in the design and fabrication of large arrays of detectors at millimeter and sub-millimeter wavelengths. Current solutions include antenna-coupled phased arrays, Si platelet lenslet horns, and Si hemispherical lenslets. In order to overcome the limits of these technologies, we have investigated and demonstrated alternative approaches based on metamaterials that will help enable future lens-coupled instruments.

The first approach, the Si-etched-holes GRIN lenslet, provided good results although it faced manufacturing limitations for the high-frequency CMB bands. The second approach, the air-gap SiN mesh lenslet, showed very good agreement with the simulations, but it is designed to couple antennas in free space. The third approach, the Si-embedded mesh- GRIN lenslets, allowed direct coupling within Silicon, a requirement for Si-based detectors. Ultimately, the upper-frequency limitation of these approaches is governed by the layer-to-layer thickness of the substrate. For the silicon-based approaches here, the 100-μm-thick layers used can operate to ~ 300 GHz, and initial work down to 75-μm-thick layers which would extend the range to nearly 500 GHz have been promising.

The next step in our developments will be to simplify the designs by reducing the number of grids and layers. This will be carried out by designing: (1) Si-embedded mesh lenslets based on phase delays; (2) lenslets with both transverse and longitudinal arbitrary GRIN patterns; (3) hybrid solutions including both phase delays and GRIN structures.
